# Designing technology to support greater participation of people living with dementia in daily and meaningful activities

**DOI:** 10.1177/20552076231222427

**Published:** 2024-01-15

**Authors:** Michael Wilson, Julie Doyle, Jonathan Turner, Ciaran Nugent, Dympna O’Sullivan

**Affiliations:** 1 NetwellCASALA, 8817 Dundalk Institute of Technology, Ireland; 2 ASCNet Research Group, Department of Computer Science, 8819 Technological University Dublin, Ireland

**Keywords:** Dementia, technology, meaningful activities, collaboration, self-management

## Abstract

**Background:**

People living with dementia should be at the center of decision-making regarding their plans and goals for daily living and meaningful activities that help promote health and mental well-being. The human–computer interaction community has recently begun to recognize the need to design technologies where the person living with dementia is an active rather than a passive user of technology in the management of their care.

**Methods:**

Data collection comprised semi-structured interviews and focus groups held with dyads of people with early-stage dementia (n = 5) and their informal carers (n = 4), as well as health professionals (n = 5). This article discusses findings from the thematic analysis of this qualitative data.

**Results:**

Analysis resulted in the construction of three main themes: (1) maintaining a sense of purpose and identity, (2) learning helplessness and (3) shared decision-making and collaboration. Within each of the three main themes, related sub-themes were also constructed.

**Discussion:**

There is a need to design technologies for persons living with dementia/carer dyads that can support collaborative care planning and engagement in meaningful activities while also balancing persons living with dementia empowerment and active engagement in self-management with carer support.

## Introduction

### Background

Living well with dementia has become a key objective of public health and research plans internationally,^1^ with the focus progressing from prolonging life to also enhancing quality of life, through delaying or preventing further disability.^2,3^ This shift also represents a move from a traditionally more medical view of dementia to a more social view, with an emphasis on well-being.^4,5^ As such, the concept of self-management for people with dementia has emerged, defined as “a person-centred approach in which the individual is empowered and has ownership over the management of their life and condition. The role of health and social care providers is to support the person's journey towards living well in the presence of absence of symptoms.”^6,7^ Further to this, practice recommendations for person-centered care have been recommended, based on current best practices, which emphasize the importance of knowing the person living with dementia within the context of an interpersonal relationship such that individualized choice and dignity are supported. At the same time, ongoing opportunities for meaningful engagement need to be identified and facilitated within a supportive community.^8^ Dementia is a progressive and degenerative disease, the main symptoms of which are cognitive limitations and degradation of everyday practical capabilities, impacting the person living with dementia's ability to access, process, or remember information.^9,10^ It is estimated that close to 50 million people globally are living with dementia and that each year there are 9.9 million new cases of dementia worldwide.^11^ Dementia shortens the lifespan of those affected, while also severely impacting the quality of life for both people living with dementia and their carers.^11^ However, there is increasing recognition that those particularly in the early stages of dementia have many years in which they can live meaningful lives while managing their condition.^6,12,13^ Moreover, recent work focusing on temporal trends in quality of life among nursing home residents with late-stage dementia has suggested that quality of life can be maintained and improved upon for this cohort, with enhanced activities and social engagement playing a role in its preservation.^14^

In parallel to this, the design of technology to support dementia has also undergone a shift, from technology focused on monitoring and supporting activities of daily living (ADLs),^15,16^ safety (e.g. preventing wandering)^17^ and supporting carers^18,19^ to empowering those with dementia to engage in decision making around care planning and self-management.^6,20^ Supporting planning and engagement in meaningful activities is one area of recent interest. A definition of meaningful activities is offered by Phinney et al.^13^ as “the spectrum of occupations a person performs in his or her everyday life and that are perceived as significant to the person.” People living with dementia are likely to perceive an activity as meaningful when it brings feelings of pleasure, enjoyment, or belonging and allows for a sense of autonomy and personal identity.^13^ Human–computer interaction (HCI) technologies to support meaningful activities have focused on reminiscence,^21^ social engagement,^22^ music therapy,^23^ and planning activities.^24^ However, the focus of such technologies on very specific activities does not align with the preference-based model for care as outlined by Van Haitsma et al.,^25^ which suggests that facilitating needs, creating the conditions for behaviors to achieve goals and indeed delivering healthcare for older adults, in general, all need to be based on an individual's values and preferences. As such, engagement in meaningful activities is still very much an unmet need for people living with dementia.^26^

There has also been recent emphasis on the importance of designing for persons living with dementia/carer dyads, given the critical role informal carers play in supporting those with dementia. Furthermore, the progressive symptoms of dementia can make it challenging for people living with dementia to adopt and engage with technology use, and therefore it is important to explore how carer support can be integrated into technology design.^24^ Traditionally, technologies for person living with dementia/carer dyads have focused on the carer as the primary user, with the person living with dementia often taking a passive role. For example, technologies focused on monitoring, safety, and security often track the person living with dementia's movements and activities, providing feedback on these to carers. However, as outlined above, people living with dementia, particularly those in the early stages of the disease, should be supported in playing a more active role in self-management and engagement in meaningful activities. There is a need therefore to design technologies for persons living with dementia/carer dyads that balance empowerment and active engagement of people living with dementia in meaningful activities with carer support. However, understanding how dyads collaborate to manage illness and care together is an emerging area of enquiry, with comparatively little research conducted to date^27^ both in the social and HCI literature.

### Meaningful activities and people living with dementia

Meaningful activities are those that focus on values and beliefs that resonate with the person living with dementia, particularly with past interests, routines, and roles.^28^ There is a large body of research on the types of activities for people living with dementia that are considered meaningful. Phinney et al.^13^ identified four types based on analysis of multiple interviews and observations of people living with dementia with mild-to-moderate impairment: (1) leisure and recreation (e.g. hobbies and going for walks), (2) household chores (e.g. making a bed and helping out with meal preparation), (3) social involvements (e.g. continued contact with family members as well as interactions at support groups with other people living with dementia), and (4) work-related activities (e.g. transferring work-related skills to everyday situations including offering guidance and advice). Similar activities have also been identified as important by others.^29^ Additionally, the importance of cognitive activities (e.g. concentration card games and puzzles), music and entertainment, manipulation/sensory/sorting (e.g. sorting jewelry), and reminiscence have been noted,^29^ and identified as activities often prescribed by occupational therapists.

Meaningful activity can be considered an important parameter of psychosocial health, and the overall benefits to people living with dementia of engaging in such activities are varied, and considerable and are derived from a broad range of everyday activities.^30^ With regard to leisure activities in particular, studies have shown various benefits including improvements in overall wellbeing,^31^ a reduction in dependence on services^32^ decreased social isolation,^33^ and deceleration in the rate of cognitive decline.^34–37^ Lower levels of depression and improved physical health have also been observed after three months among people living with dementia engaged in higher levels of meaningful activity.^30^ Importantly, engagement in meaningful activities can also result in a feeling of belonging and a sense of self-identity,^13^ as well as the promotion of autonomy and the potential for a reduction in the amount of dependence on family members or carers.^38,39^ Engagement in meaningful activity has been found to not only benefit the person living with dementia, but has been shown to reduce both carer burden and time spent on caring in general.^40^

More recently, work into the development of the Meaningful and Enjoyable Activities Scale (MEAS) in mild dementia found there to be five independent themes surrounding the importance of engaging in meaningful activity.^30^ These included (a) retaining mastery and experiencing agency, (b) providing opportunities to compensate for age-related losses and losses associated with dementia/cognitive impairment, (c) allowing for continuity for the individual's roles and “life story,” (d) connecting with others, and (e) mobilization of resources to cope with age-related or dementia-related changes.

### Technologies for people living with dementia and carer dyads

A vast array of technology has been designed to support people living with dementia and their care. The majority of such technology is focused on monitoring, security, and safety. Several studies have focused on using in-home and wearable sensors to monitor activities of daily living, to detect changes in patterns of behavior that might be indicative of disease progression.^15,16^ Technology research has also focused on the detection of wandering,^17^ and agitation.^41^ The primary user of these types of technologies is typically the carer, while the person living with dementia is often a passive user. However, some research also strives for a more inclusive approach, engaging the person living with dementia in tasks where monitoring is employed. The Ambient Kitchen^42^ makes use of an array of sensors and cameras in the kitchen to guide the person living with dementia through simple food preparation and cooking tasks. Other research aims to facilitate safe and independent outdoor walking through the use of sensors and AI to nudge the person living with dementia via a sensor interface to support them in returning home independently, prior to alerting a carer.^20^

While these technologies may become increasingly important as the disease progresses and people move into the moderate to severe stages of dementia, technology should also strive to prolong the period of independent living for the person living with dementia and to support the maintenance of quality of life, for example, through promoting engagement in meaningful activities.^43^ Technologies focusing on meaningful activities can be categorized into four main purposes: reminiscence/memory support, for example,^21,44–49^ behavior management, for example,^50–53^ stimulating engagement, for example,^54–58^ and conversation/communication support, for example.^46,59^ Recent research on people with mild to moderate dementia who use various technological supports in their self-management of their condition has demonstrated the capabilities of people living with dementia to self-manage, with the authors concluding that “people with mild to moderate dementia are inventive creators and capable actors in self-management.”^60^

However, it is well understood that informal carers are core to supporting self-management for people living with dementia, particularly given the progressive nature of the disease. The role of informal carers in providing care, support, and quality of life for older people with dementia has become valuable in today's society. The estimated number of hours of informal care (ADL support and general supervision) provided to people living with dementia living at home was 86 billion in 2015, which equates to approximately 6 hours per day per person living with dementia, 60% of which is related to ADLs.^61^ The demands associated with providing full-time and unpaid care are considerable and varied, frequently resulting in feelings of isolation, psychological distress (anxiety, depression, and stress), loss of self-esteem, and a tendency to neglect one's own health and wellbeing due to time constraints and feelings of exhaustion.^62^ These issues can then negatively affect the carer's capacity to provide adequate care, increasing the likelihood of negative health outcomes for both the carer and the care recipient.^63^ As such, various HCI research has explored technology to support carers and a wide range of technological solutions now exist, including online support tools or educational programs,^19,64^ digital interventions to address burden, stress, anxiety, and depression,^6,65,66^ solutions aimed at social connection and peer support,^67,68^ as well as those more focused on supporting the carer to self-manage their physical health.^1^

Comparatively little research has focused on technology use by dyads. Studies have found that the successful integration of assistive technology depends not only on the person living with dementia; the carer must also find it interesting and engaging.^69^ It has been emphasized that even those people living with dementia at an early stage of dementia may not be able to manage technology if they are living alone and are not in receipt of support from an informal carer or relative.^70^ Unbehaun et al.^4^ note the importance of the carer for motivating initial engagement with the technology, with the person living with dementia then willing to engage with further interaction. Similarly, a study exploring the use of the Google Home conversational agent with people with mild cognitive impairment (MCI) and carer dyads found that MCI makes it challenging for people living with dementia to even remember that the technology exists and is available within their homes.^71^ Carers in that study spoke of initiating strategies to prompt engagement by the person with MCI, such as printing laminated cheat sheets and instructions on how to use the technology and placing these within visual reach of the technology. Furthermore, carers implemented scaffolding to minimize the complexity of interaction by participants with MCI, for example through setting up reminders and calendar events through the technology that could be targeted to the care recipient.

However, benefits have been identified for both people living with dementia and carers in studies exploring dyad technology use. Carers perceived that structured activities, such as participating in exergames, relieved boredom for the person living with dementia, and motivated them to engage in exercise, in addition to resulting in improvements in activity and fitness.^4^ Furthermore, the time spent engaged in the exergame activity by the person living with dementia provided time for the carers to engage in other activities, such as having a friend visit.^4^ At the same time, technology designers need to be mindful of the possible negative consequences of technology use by people living with dementia and carers. One study illustrated that technology can both promote and reduce stress for informal carers, while both increasing feelings of autonomy and feelings of incapacity for people living with dementia.^69^

### Research aims

Our goal was to explore issues around maintaining quality of life, while also gaining a greater understanding of the importance of collaboration and shared decision-making between people living with dementia and their informal carers around daily and meaningful activities. Related to this was an exploration of how carers could be supported to encourage care recipient participation and engagement. Through this, we aimed to develop design recommendations when designing technology with and for people living with dementia to support engagement in activities.

## Methods

Below, we outline procedures including recruitment, demographics, and interview protocols, as well as our approach to data collection and analysis. Ethical approval for the study was received from the research ethics committees of both the institute of technology and the regional health service. The COREQ guideline for reporting qualitative results was used and is provided as the Supplemental Material.

### Procedures

*Recruitment.* We aimed to recruit individuals with a diagnosis of pre-dementia MCI or early-stage dementia. These diagnoses were based on referrals to dementia support services (as outlined below). Participants were recruited through two sources: PLwD1 was recruited though Alzheimer's Society Ireland, while the other people living with dementia and the ICs were recruited through a local outpatient referral center for older adults. Recruited occupational therapists (OTs) all worked at the same outpatient referral center, which specializes in assessing and managing cognitive impairments, the most common types of dementia, frailty syndromes, as well as a variety of other diseases associated with aging. The purpose of the center is to provide a multi-disciplinary assessment for patients, and onward referral and signposting to community services. The team includes Geriatricians, Clinical Nurse Specialists/Advanced Nurse Practitioners, OTs, Physiotherapists, and Administration support. The Dementia Case Manager (DCM) was the case manager for the local area for the last 7 years.

Information leaflets describing the study were provided and clinicians made the first approach to potential participants. The aims, procedures, risks, and benefits were outlined, and the clinicians provided an overview of the study and gauged interest. It was also made clear that participation was entirely voluntary and independent of any clinical care plan and that withdrawal without prejudice at any time was possible. The research team checked in regularly with staff to determine interest. If a potential participant wanted to know more, a member of the research team called them and their carer to discuss the study in more detail. Those who expressed a willingness to volunteer for inclusion were asked to give written informed consent and were asked for permission to include their informal carer in the study. However, this was not a requirement to take part.

Participants needed to be able to understand the study requirements and give informed consent. Researchers obtaining consent were guided in particular by issues around informed consent and capacity to participate. Older people, especially those living with cognitive impairment are a vulnerable group, and safeguarding their rights was a key concern. Clinical staff excluded those they considered unable to give informed consent or those for whom the study would have been likely to prove too demanding for either the person living with dementia or their carer. Our aim was to allow for participation wherever possible. However, it was vital that we were guided by the clinical specialists as to when informed consent could not be guaranteed. Given that the participants were referred and not recruited directly by the authors, information on how often exclusion was the case was not available.

*Protocol.* The interviews and focus groups were specific to individual stakeholder groups such that the persons living with dementia/carer dyads were not present in health professional (HP) interviews/focus groups and vice versa. Following a review of the literature, topics explored with participants included what constitutes quality of life for people living with dementia, how to maintain quality of life over time, the relationship between engaging in daily activities and maintaining quality of life, and which activities bring meaning and why. Questions were also asked about activities that were valued and enjoyed before the dementia given it is reasonable to consider such activities are intrinsically meaningful in terms of everyday life and past experience.^13^ Interviews also explored the goals of participants in relation to future health and wellbeing. The same interview protocol was used with both the dyads and the HPs, covering the same topics overall but with adaptations made to accommodate the profile of participants and their perspectives in terms of lived experience regarding living with dementia, caring for people living with dementia informally, or working in a professional healthcare capacity with people living with dementia. The interviews and focus groups with healthcare professionals provided a clinical perspective of what is important for self-care and care planning for people living with dementia and their carers. The interview guides were discussed with the research team prior to deployment and further refined. Finally, public–patient involvement (PPI) volunteers (persons living with dementia and informal carers) from the Alzheimer's Society provided feedback and guidance on the protocol and participated in pre-tests. The interview guide is provided as the Supplemental Material.

### Data collection and analysis

Semi-structured interviews were held with four people living with dementia/carer dyads in the participants’ homes lasting ∼ 60 minutes. PLwD1 was interviewed on three separate occasions overs Zoom without his informal carer present, with each interview lasting ∼ 45 minutes (this format was used due to COVID-19 restrictions being in place at the time) Table 1. Participants had the option to participate in the interview with their partners or carers; all opted to do this with the exception of PLwD1. Carers were three spouses and one daughter who lived with the person living with dementia for part of the week. The interviews were conducted between August 2021 and April 2022. Separate semi-structured interviews were held in person with a DCM on two separate occasions, with each interview lasting ∼ 90 minutes. Four OTs took part in two separate focus groups held over Zoom, each lasting ∼ 90 minutes. The semi-structured interview with the DCM and the focus groups with the OTs were held between September 2021 and December 2021. All data collection was conducted by the first author, who is a post-doctoral researcher with experience in digital health, health, and well-being self-management for older adults and qualitative research.

**Table 1.** Participant demographics: PLwDs and ICs dyads.

Participant ID	Age (years)	Gender	Dementia type	Relationship to IC
PLwD1	58	Male	Lewy-Body	n/a
PLwD2	79	Male	Alzheimer's	IC2 (wife)
PLwD3	87	Male	Alzheimer's	IC3 (daughter)
PLwD4	79	Male	Alzheimer's	IC4 (wife)
PLwD5	75	Male	Vascular	IC (wife)
IC2	74	Female		
IC3	n/a	Female		
IC4	73	Female		
IC5	75	Female		

Interviews and focus groups were audio recorded using a dictaphone. Prior to analysis, the recordings were transcribed and participants were given ID numbers. The transcripts were imported into NVivo (qualitative data analysis software) for analysis. Inductive thematic analysis was employed, using the six-step approach outlined by Braun and Clarke,^72^ to provide a framework for identifying the themes or patterns within the data. Following familiarization with the data (step 1), line-by-line coding was employed separately by two researchers (the first and second authors) to allow for critical reflection and minimize the potential for individual bias as well as existing assumptions and values shaping the data interpretation (step 2). The first and second authors then held re-coding workshops to agree on the codes and their structure and to identify patterns of meaning in the data related to the research questions by collating and grouping together codes, which were then developed into themes (step 3) and further refined into sub-themes through continued analysis and discussion by both researchers (step 4). Themes were identified at a latent level, focusing on “underlying ideas, assumptions, and conceptualisations” (Braun and Clarke^72^^(p.84)^), with the development of themes and patterns based on interpretative work by the researchers (steps 5 and 6). A coding example has been provided as the Supplemental Material.

In order to ensure the trustworthiness of the data, four primary criteria were used credibility (through prolonged engagement with the data and data collection triangulation), transferability (the findings can be applied to similar contexts or individuals), dependability (the process was documented throughout to allow for another researcher to follow the decision trail), and confirmability (in order to ensure that the researchers’ interpretation and findings are derived from the data, an audit trial in the form of a coding schema was used, outlining the codes and patterns identified in the analyses).^73^

## Results

### Demographics

#### Overview of themes

Inductive thematic analysis of the interviews and focus groups allowed for the construction of three themes: (1) maintaining a sense of purpose and identity; (2) learning helplessness; and (3) shared decision-making and collaboration. Within each of the three main themes, related sub-themes were also constructed (see Table 2).

**Table 2.** Themes and related sub-themes.

Theme	Sub-theme
Maintaining a sense of purpose and identity	The impact of dementia on sense of self
	Strategies for maintaining purpose
	Identifying future meaningful activities
Learned helplessness	Fear and increased dependence
	Harming when trying to help
Shared decision making and collaboration	Shared decision making in care planning
	Finding opportunities to share tasks
	Working together to try new things

#### Maintaining a sense of purpose and identity

The importance of maintaining a sense of purpose and identity following a dementia diagnosis was discussed by all participant groups. Sub-themes related to the impact of dementia on one's sense of self, strategies implemented for maintaining purpose, and identifying future meaningful activities. Emphasis was placed on how important maintaining a sense of purpose and identity can be to prolonging independence, ensuring participation, and facilitating empowerment.

*The impact of dementia on the sense of self*. For many people living with dementia, the effects of the disease on one's independence and ability to do things you once did or want to do can be challenging. PLwD5 discussed no longer being able to cook: “that has been taken out of my hands”; while PLwD2 spoke of how his level of engagement in certain activities had dropped: “there are a lot of things I want to get going for a while now and I just wasn't capable of doing it … I lost mobility and the interest in doing the google searching. I just got lost a bit as I said I had a bad experience.” A significant blow to PLwD5 was being advised he could no longer go running, which was his primary interest. He explained that “the running was big … I had been so used to doing it for so long and then it's been taken away from me.” Retaining a sense of identity, such that the individual does not feel automatically associated with being someone who is cared for or simply a person with dementia was very important to some participants. For example, PLwD 1 recalled being in a local pub with some friends and the sense of normality he felt, saying “I was just PLwD1, one of the boys having a laugh, having some fun.”

The HPs also explained how instances such as these can negatively impact mood. HP2 said “there is a high instance of low mood and that can be quite closely linked to people feeling that they don't have any control,” while HP1 commented that “it's just that they have been consigned to ‘you’re useless, you are too slow’ … so what does that affect?, your self-esteem.” Since he was advised not to drive, PLwD5 felt “very isolated.” For those participants who can still drive, it was something they considered to be very important. PLwD2 said “I am still driving … I’d hate not to be able to do that … I’d be worried about losing the driving licence because I like to be independent.” The inability to drive can have particularly negative consequences for those living rurally, typically leading to social isolation. IC4 described the lack of opportunities for social interaction in their area, saying “in country areas it's very limited.” HP3 explained that rural isolation often resulted in people not being able to access services. She said “They couldn't get a lift. They lived too far away. They didn't have … local bus routes running past the houses or they didn't want to bother their families and to consecutively do it for like seven weeks, a lot of the time they felt that it was too much.” HP2 stressed that it is important for people in such situations to plan accordingly in order to be able to get from place to place, saying “it becomes more about a person planning their, their week more and that they know what they need to do in the week.”

*Strategies for maintaining purpose.* All stakeholders spoke about the importance of implementing strategies around engaging in daily tasks and meaningful activities, for maintaining a sense of purpose, independence, and quality of life. The need to identify everyday activities and establish household roles and responsibilities at an early stage was highlighted by HPs. HP1 stressed, “the first thing is to keep doing everything you do, you can do for yourself.” She explained that household tasks should continue in the same way wherever possible, saying “If your job was always kettle, make the porridge and the other person set the table, that task should remain the same.” The same should apply to everyday activities outside the home according to HP5, who said “if it's working for you now and there are no big issues and you are not getting disorientated when you are out, then keep going.” The person living with dementia perspective was similar. PLwD1 spoke about continuing to meet friends in his local pub*,* while PLwD5 expressed how important it was for him to stay fit, saying “If I could keep up my walking, my exercises … I just want to keep myself right.” Similarly, PLwD4 spoke about trying to maintain cognitive health through reading and meditating. He explained that he was “worried that maybe my brain activity is not as good as it should be and I might be going down and I am trying to constantly reading a bit and slowly to keep it up.”

The value one feels from having engaged in a task was emphasized by HP1, who provided the example of someone who likes sweeping the floor. She said “your answer is well then every hour you say, ‘would you mind sweeping the floor, just give it a quick run round’. Every hour you do that is of value … it's empowering them.” This sense of empowerment that comes with such participation also extends to driving, provided the individual is still competent. HP1 explained, “facilitate where the person is at … If a person can drive, I would say to the partner, don't you drive … Let them drive if you feel comfortable sitting in the car.” These sentiments were echoed by the people living with dementia, with PLwD4 (who can drive) saying a good quality of life involves being mobile “without having to depend too much on outside help,” and PLwD1 (who no longer drives), saying “I’m caught that way. Like I cannot go anywhere on my own, I need help.”

HPs stressed the importance of PLwDs having a routine, with HP1 saying “people who do well with dementia are people who actually stick to a routine.” A daily schedule was also described as very useful, with HP4 saying “the person can regularly refer to it throughout the day and then … they can tick it off, then say “the next thing on my daily list is to have my dinner or to have my lunch.” Conversely, there is also potential for one's day to become repetitive, monotonous and boring, sometimes leading to feelings of frustration or consistent low mood. IC5 commented: “We have a very boring life and that is no good….” PLwD5 explained further that it is difficult to adjust to this new situation as they used to live busy lives (IC5: There weren't enough hours in the day. PLwD5: And now there's too many). This can have a negative impact on mood. IC5 explained how she feels her husband becomes “tired of life” in general, while he responded that “I find it so frustrating at times I could scream.”

*Identifying future meaningful activities.* HP3 emphasized the importance of finding “what's meaningful to them, in terms of what goals they would like to achieve or what they would like to participate in.” PLwD1 spoke about finding things to appreciate, saying “a good quality of life for me now means being able to enjoy the day.” Identifying interests and finding the right activities to stimulate these interests now and into the future was considered important. Frequently these interests related to what the individual enjoyed doing throughout their lives. HP5 explained that “generally it will stem from what their working life was like or their roles and responsibilities and the family or, what their hobbies were years ago or currently.” The role of the family in facilitating activities was also discussed, with HP4 saying “it's educating them around that and maybe supporting the family to … have a programme throughout the day.” As well as this, it is important to recognize that interests and goals are unique to an individual and will vary from person to person. HP2 said “it's whatever is important to them … and then you prioritise on that basis.”

However, as outlined above, over time people living with dementia can lose the ability to engage in activities that are meaningful to them. The importance of identifying the right replacement activities was stressed by HPs but this was not something that PLwD participants had much success with. For example, PLwD5, whose main interest prior to having a stroke was running, has not been able to find an effective substitute. IC5 explained that “the walking has nothing for him, the exercise bicycle has nothing for him, the rower machine has nothing for him.” Again, PLwD5 voiced his frustration at his situation, saying “I’m just frustrated that I can't do … to me I can still do it but I know I can't because I know … if I went out for a run I’d end up in the ditch.” However, this was not the case for PLwD1, who explained that in instances where he can no longer do something, he will try to find an adequate replacement He said “rather than beating myself up about it, I will try and get something to replace it. I will try and get something else to look forward to.” HP1 discussed the importance of finding replacement tasks. She said “it's awfully important then to have another task, so what's the other task? What did they do? What did they engage in?” She provided the example of a person who can no longer play golf. One option would be to use a set of golf clubs in order to stimulate conversation and reminiscence. She said, “the memories that will kick off in him that you know nothing about is invaluable.” She explained further how this is “a version of the activity that was.” For other activities that someone is not engaging in but may again be possible, HP5 explained that is important to “grade the activity to a level they can then return to doing it.”

The need to address cognitive decline and find appropriate and effective sources for cognitive stimulation was also widely discussed. The PLwDs discussed different techniques and sources for cognitive stimulation, with PLwD5 enjoying playing solitaire, and PLwD1 playing mobile golf games, which he feels “gives me a barometer in the morning of where I am at.” IC4 discussed using digital photo frames with PLwD4 as a means of stimulating conversation and reminiscence: “remember we did that, or … do you know which of the children that is now? When they were little?” Similarly, she follows local Facebook groups that share old photos of their local area and uses these to help PLwD4 reminisce, particularly about football. The OTs also discussed using reminiscence as a means of cognitive stimulation during their group sessions with PLwDs. HP2 said, “we would generally bring in the newspaper from that week … ask people to talk about the news topic … challenging their orientation, their memory skills, their executive function.” It was also suggested that activities which are new to the individual can also be very effective. HP3 said, “we always try to encourage new learning, new involvement and new activities and that's when your brain is working its hardest.”

#### Learned helplessness

Both the people living with dementia and carers spoke of the fear they felt as a result of the dementia. Feelings of anxiety and general fears for the person living with dementia's safety were evident. HPs also spoke of the fear that people living with dementia experience in relation to making mistakes as a result of their illness, highlighting the impact this can have on both confidence and relationships. Such fear appears to result in an increase in the person living with dementia's dependence on their carer and the carer striving to do as much as possible for the person, the outcome of which can be that the person living with dementia develops a sense of learned helplessness.

*Fear and increased dependence.* The fear that comes with dementia can be difficult to understand as it is often not associated with any reason or source in particular. PLwD1 commented that “normally if anybody is afraid obviously you can say I am afraid because they think something bad is going to happen but this is a generalised anxiety.” Outings that would seem simple, enjoyable and safe can appear very frightening for a person living with dementia; this is especially so if their primary carer is not present. PLwD1 provided an example of going for a drink with his brothers without his wife present, saying. “I reluctantly went. I was terrified. My brother asked ‘why are you so afraid?’ I said ‘I don't know’.” He continued “I am getting to the stage in my dementia that this is my safe place.” Similar sentiments were expressed by HP1 who explained that “they are kind of slightly afraid of everything that is bigger than in your own house.” She also explained that living without fear is crucial for good quality of life: “If you are afraid, you are anxious. Your quality of life is very poor because it has knock on effects into your relationships, your diet, your exercise, your everything.” HP1 suggested that a person living with dementia knowing their IC is aware of their struggles and is there to support them can bring about “huge piece of mind,” and the outcomes can include “your quality of life is great. Your mood is better so your activity levels are generally better. Your social interactions are better.”

The frequency, significance, and possible ramifications of the types of “mistakes” being made by people living with dementia were discussed. HP1 pointed out that someone with MCI or early-stage dementia will be aware of impairments themselves and recognize they are making mistakes “before anyone else notices.” A huge source of concern from someone with MCI is how their loved ones will react to mistakes becoming apparent once they communicate the problem to them. Trust is therefore hugely important, and people living with dementia need to feel comfortable in revealing mistakes made and communicating how they are feeling with their IC. A person's personality will often decide whether they continue to cover up mistakes or if they reveal this to a loved one, which can persist as a huge conflict in the mind of someone with MCI or early-stage dementia. HP1 said, “do I feel safe enough to admit that I am making mistakes and hope they will understand and work with me and that's the huge pull and push.”

The degree to which people living with dementia have become dependent on their informal carers was also discussed by all stakeholders, with this often being linked to a lack of confidence and fear within both people living with dementia and ICs. Both PLwD1 and PLwD3 described not being able to go anywhere unaccompanied and feeling a sense of security at having loved ones nearby. PLwD1 said “I cannot go anywhere on my own,” while PLwD3 explained: “In case I would stagger or fall or anything … I am not as confident*.*” This sentiment was echoed by both ICs and HPs. IC5 explained that “Mass is important to him. I go because it's the walk up and I’d be afraid anything would happen to him that he just wouldn't make it up and back.” HP1 mentioned that a sense of security and feeling safe can be crucial for PLwDs, saying “There is just security in the other person being with you and feeling ‘well I am safe once there is another responsible adult with me’.’’

*Harming when trying to help.* As a consequence of a carer trying to both address mistakes and make the person living with dementia feel safe, the outcome can be the phenomenon of harming when trying to help. Noticing and worrying about mistakes can lead to “overhelping” such that an entire activity is taken out of the person's hands, when it is possible that the ability to carry out certain parts of the activity may still be intact. HP3 spoke of family members dropping groceries into their relatives. This can in turn lead to the individual no longer walking to the local shop and exercising the various skills and functions required to do this, which can be vital in terms of both physical exercise and social interaction. She said, “is it that their family are just overdoing it for them? … they are over-compensating … often you get a lot of anxious family members and … they think that they are doing the favour.” Similarly, HP1 explained that informal carers will frequently try to complete household tasks while the person living with dementia is still in bed, but that this can have negative ramifications. She said, “it's actually pushing the person … into a consistent low mood because now they have had three or four extra hours in bed, they have had no activity … They are now 50% of their day in bed.” HP2 explained that it is important to establish the degree to which someone is performing a task for the person living with dementia, and if there are any levels of participation. She said “you have to delve into what exactly are you doing? Are you doing the full task part of the task? Is there any piece that the person is doing themselves?” The OTs also pointed out that the level of support provided needs to be carefully balanced. HP2 said, “it should be enabling support as opposed to a disabling support and you should be trying to maintain function rather than totally taking that away.” Indeed, some examples of this were apparent in the dyad interviews, whereby the IC at times responded on the person living with dementia's behalf and corrected their mistakes. While difficulties articulating or remembering information are to be expected among this cohort, balance is needed in order to prevent the person living with dementia from becoming more and more passive in their involvement, inclusion, and engagement in conversations and activities in general.

#### Shared decision making and collaboration

The final theme concerned the role of collaboration and shared decision-making in living well with dementia, and the importance of education in supporting this.

*Shared decision-making in care planning.* When designing a care plan and outlining goals for daily activities, HP2 stressed the importance of working together with the person living with dementia and their IC, saying “it is a collaborative process with the patient, their carers you know it has to be very well thought out and it has to be very well consented in the care plan.” HP4 noted that goals in particular are individual and personal and should not be assumed without consultation with the PLwD. HP2 continued “because I might identify a goal and they will say that is not important to me. What is important to me is this instead and then it might totally change the intervention.” As well as this, the person living with dementia must be able to find meaning in what they do, without having activities set for them, as explained by HP4: “(we are) making sure that a person can do tasks and activities that they find meaningful and purposeful, not something that we would prescribe for them, that they would be engaged in that process and have to be accepting of it.” It became apparent from the discussion with the OTs, that in order for activities to be engaging, cognition stimulated, and function maintained, family education is required and a collaborative process involving the person living with dementia must be followed. HP3 said, “maybe it becomes a bit more about them as opposed to the person and what they think the person can and cannot do and there is a huge education piece to the family.”

*Finding opportunities to share tasks.* Another way in which the importance of collaboration was emphasized concerned sharing tasks. Being able to do even a small part of a particular ADL or meaningful activity should be recognized and maintained wherever possible. The area of household roles and responsibilities and how these are either maintained or altered following the onset of dementia was discussed with each group. People living with dementia and their ICs discussed how tasks are shared within the household. PLwD5 said “she normally makes out the list but I push the trolley,” while his wife said, “we make the bed together, we put the clothes on and the duvet cover.” Similarly, IC4 explained how meals are prepared together, saying “he would help me prepare the vegetables to make the soup; this was the same for other household tasks: it would be joint … laundry I would … organise it … he would hang out the washing.” It became apparent that the informal carer can assume a new role within the household, often to the detriment of the person living with dementia's morale or confidence. This removal of responsibility and place within the household can have a significant impact and even lead to feelings of uselessness or worthlessness. For example, IC5 explained that she now manages finances, which normally would have been the responsibility of PLwD5. She said, “he doesn't like that, not now he doesn't like that because like I say it's taken away from him.” This was also the case for PLwD2, who explained his wife was “taking care of that this last while … because I went through quite a bad spell … I don't know what happened but I really wasn't able.” While collaboration between people living with dementia and ICs is important, the previous theme outlined the potential negative impact of “over-helping.” HP1 emphasized the importance of engaging the person living with dementia in such household tasks for self-esteem. She also recommended certain strategies to increase participation, saying “if you are in the house and the person is doing nothing, you could say ‘well my back is sore, would you ever be able to carry that laundry in here?’” However, she also pointed out that completing the task should not be the aim, suggesting “attempting the task is all that counts.” Similarly, the OTs stressed that it is crucial for informal carers to retain a sense of balance in terms of the amount of support they provide, taking a step back at times and recognizing that there is a level of acceptable risk associated with certain activities. HP2 noted that ICs should “break down the activity and allocate whatever proportion of the activity they are able to do.” She also highlighted that “it can be important to reassure the carer that it's ok, this is an acceptable level of risk.” HP1 pointed out that it is important to allow for the sufficient time needed for such participation, saying “they cannot think that fast … stop the rushing.”

*Working together to try new things.* As well as establishing a person's interests and personal goals, the HPs also stressed that it is important for PLwDs to try new things. Educating both the individual and their IC about the potential benefits in terms of other skills and functions can be an effective way to encourage engagement, with HP3 saying “you explain to them the importance of it then they generally tend to be a little bit more open.” This was echoed by HP2, who explained that “you're supporting someone to go outside their safety net and you know encourage them to push the boundaries of what their abilities are.” Examples were also provided of activities that can exercise multiple skills and functions, which can be recommended to people living with dementia. HP4 explained that something as simple as hanging clothes on a line has multi-sensory benefits for people living with dementia, saying “the smell from the fresh clothes, you have the physical aspect of reaching up and putting them on the line and then the visual aspect of the locality so it is quite encouraging.” HP1 also suggested parking in the furthest space from the entrance to a supermarket to encourage physical activity: “Go up and get your trolley. Go in, go around, come back out. Walk back over with your trolley. That is one of your walks for the day done.” Other tasks such as bringing laundry up and down the stairs were also recommended. In terms of meaningful activities, examples such as dancing (sequencing and processing: HP2), yoga (“planning and organisation even of movements and coordination”: HP2) and swimming (“for your muscles but also your mental health”: HP2) were provided to highlight how activities that might be new to someone can be beneficial in a range of ways.

## Discussion

The findings outlined are from focus groups and interviews with persons living with dementia/carer dyads and HPs involved in their care. In total, 14 participants took part, resulting in a rich, qualitative data set for analysis. Three related themes were identified in the data, which center on the need for people living with dementia to remain engaged in daily activities wherever possible with the support and collaboration of carers and/or family members where necessary. Our findings show how dementia has a significant impact on multiple aspects of an individual's quality of life, negatively impacting their sense of self and sense of purpose. As evident in our findings, as well as the literature,^74^ involving the person living with dementia in planning and executing meaningful activities and encouraging them to state their preferences in this regard can positively impact a sense of purpose as well as quality of life for both the person living with dementia and carer.^27,30,40^ However, carers often underestimate the importance people living with dementia place on care values and everyday preferences, and this can lead to strains in the person living with dementia–carer relationship. Furthermore, our findings also indicate that both PLwDs and carers are often risk averse due to fear and anxiety related to dementia, which can ultimately lead to carers overhelping and the person living with dementia experiencing a sense of learned helplessness, which can limit their participation in planning and carrying out activities. Indeed, engagement in meaningful activities is still an unmet need for people living with dementia.^75^ Our findings include strategies on how to encourage greater participation by people living with dementia, such as educating carers on the benefits, finding opportunities to share tasks, avoiding overhelping, planning the week ahead as well as planning for the future. Below we suggest how technology might support these strategies, encouraging participation of the person living with dementia in planning and executing daily and meaningful activities while balancing carer support.

### Key design considerations for greater participation by people living with dementia in daily and meaningful activities

*Design Recommendation 1: Educate people living with dementia and carers on the benefits of maintaining engagement in activities for as long as is possible, with clear instructions on how to achieve this.* Research has highlighted that individuals in the early stages of dementia have the potential to live meaningful lives as they progress through their illness and are in a position to manage their condition.^6,12,13,30^ However, our findings show how dementia can have a significant impact on one's sense of self, with the loss of independence that comes with no longer being able to do the things you want to do affecting one's sense of identity and purpose. While people living with dementia and carers in our study appeared to collaborate well in household tasks, such as laundry or grocery shopping, the people living with dementia also spoke of tasks or activities being “taken away” from them. This was often as a result of fear for the person's safety, or trying to support them. HPs in our study highlighted that family education is crucial to prevent carers and family members from “overhelping” and removing responsibilities from the person living with dementia. The literature has highlighted that the benefits of engagement in daily activities, be they ADLs or those activities considered meaningful to the individual, are considerable and can improve psychosocial health. Meaning is found for the person living with dementia when activities they engage in are not only perceived as pleasant and enjoyable, but also when such activities bring about feelings of personal identity and a sense of autonomy.^13^ Benefits to the carer in terms of reduced burden and more free time have also been highlighted,^4,40^ while greater focus on dyadic collaboration can also positively impact the carer’s quality of life.^27^ It is important, therefore, that at the early stage of the disease, both people living with dementia and carers are aware of these benefits and are provided with relevant and accessible sources of information and education promoting the benefits of maintaining engagement, as well as practical and efficient strategies for using this information to facilitate such engagements. Technology systems that aim to promote the participation of people living with dementia in planning and executing activities should include such education to motivate active engagement. Specific content or interventions may be required to help people living with dementia and their carers overcome the fear of continued participation in certain tasks, an issue identified in our findings.

*Design Recommendation 2: Consider how technology can support collaborative care planning and engagement in meaningful activities, while balancing carer support with the empowerment of the person living with dementia to engage.* Collaboration and shared-decision making were highlighted in our findings with regard to both the identification as well as the planning, grading, and implementation of tasks, activities, and goals. Supporting people living with dementia and their carers to implement strategies to maintain a sense of purpose through identifying their interests, and determining appropriate activities to stimulate these interests while also considering how to account for changes in ability that accompany disease progression are key areas for technology designers to focus on. Indeed, when high importance is placed on the person living with dementia maintaining independence and autonomy, people living with dementia report higher levels of collaboration and shared decision making in care planning.^67^ The HPs emphasized the need for dyads to work on this together and to build consensus as to which activities will be attempted, and to what degree the person living with dementia will participate in them. Recently, the concept of goal-oriented cognitive rehabilitation has emerged as an intervention aimed at improving functioning for people with early-stage dementia.^76^ It promotes people living with dementia, their carers, and a therapist identifying meaningful and personally relevant goals related to everyday activities. Furthermore, a randomized controlled study found that people living with dementia were capable of identifying meaningful goals and were motivated to attain them.^76^ Various categories of activities have been identified within the literature as being meaningful to people living with dementia^13,29^ and were reiterated in our findings by all stakeholders, for example connecting with others, engaging in leisure and recreation activities, and collaborating on household chores. These can provide a basis for dyads reflecting on what activities are meaningful to the person living with dementia at home particularly where therapist support may not be available. For example, an app might present a menu of ADLs and meaningful activities, with education on the benefits of each type of activity, from which the dyad can decide which activities to add to a care plan. Activities can serve a dual purpose of providing meaning while also supporting a person living with dementia in maintaining other aspects of health and wellbeing. For example, some of the strategies suggested by the HPs in our study involved tasks that combined ADLs with physical exercise.

HPs in our study highlighted the importance of having a structured daily program of activities. This could help to alleviate the daily boredom experienced by people living with dementia and their carers as identified in our findings and by others.^4^ Thus, each week a prompt could be sent to both people living with dementia and carers to develop their weekly schedule of activities. Activities could be further broken down into tasks, with some potentially being individual to the person living with dementia, while some might be collaborative. As noted above, people living with dementia and carers all appeared to have workable systems in place for sharing common household tasks, therefore applying this logic to other more meaningful activities should be acceptable to dyads. Suggestions could be made as to who could complete tasks, based on the current abilities of the person living with dementia that could be detected using combinations of sensor data, self-reported wellbeing, and artificial intelligence. Throughout the week, this could be presented as a digital daily checklist. Engagement in some tasks could be monitored by sensors, as proposed by^43^ for example, engaging in cooking activities in a similar manner to that described in the Ambient Kitchen,^42^ while engagement in others that might be more difficult to sense could be self-reported by either the person living with dementia or carer. As noted by HP1 in our study, what's important is engaging in the activity, not necessarily completion. Therefore, recognition should be provided for attempts made.

There are numerous benefits to supporting people living with dementia to engage in technology-mediated self-management, not least the potential to maintain a positive quality of life and to delay disease progression.^4,6,9^ However, carers also play a crucial role in supporting this self-management. It is of paramount importance that designers therefore seek to understand and consider the role carers play in either supporting and empowering the engagement of the person living with dementia with technology, both in the early and later stages of the disease. Many studies have noted the importance of the carer in promoting people living with dementia's engagement with technology, as well as strategies used to support this. Unbehaun et al.^4^ pointed out that initial engagement with technology is frequently made possible through carer encouragement and motivation, while Zubatiy et al.^71^ found that carers use strategies to facilitate continued engagement and use scaffolding to minimize complexity. Therefore, technology designers need to promote strategies to support engagement in activities and or technology, once that initial engagement has been initiated by the carer. This notion of enabling support as opposed to disabling support was highlighted by the HPs in our study, and the risk of developing learned helplessness is something that again needs to be considered when designing for dyads. Acceptable risk is something that needs to be assessed for certain activities to be encouraged and maintained, and the possibility for compensating for previous mistakes to lead to eventual “overhelping” needs to be recognized, which relates once more to the need for raised awareness though information provision and education.

*Design Recommendation 3: Prepare for the future—design systems that adapt to the progression of dementia.* As dementia progresses, cognition declines and symptoms in general deteriorate. Issues around technology acceptance, adoption, and continued engagement are likely to arise, further emphasizing the role informal carers will likely need to play in order for the benefits of any technologies to be fully realized.^24^ For example, initial use and interaction by the person living with dementia may develop into co-use by dyads, and eventually become more centered around the carer interacting directly with a system. Furthermore, the ability to carry out activities can be lost as time progresses. However, even at these later stages, support should be provided to ensure replacement activities are recommended and that these are both engaging and cognitively stimulating. It has also been noted that self-management systems become more important as dementia progresses, with the need to prepare for this at earlier stages.^60^ Technology designers could also consider how to adapt or customize designs across different stages of the disease, for example through increasing accessibility or considering the use of a conversational agent to minimize physical interaction.^71^

HPs in our study suggested that one approach to finding meaning and remaining engaged is to ensure there are varied sources of and opportunities for cognitive stimulation, with strategies such as reminiscence, gamification, discussion around current affairs, and the challenge of trying something new serving as examples of how this could be achieved through personalized technology support. Indeed, as outlined in the “Methods” section, much HCI research has already explored the design of such interventions. Our findings also highlighted the importance of replacement activities and the role they play in addressing situations when continued engagement in activities considered meaningful to an individual is no longer possible or feasible. If, as discussed in the previous design recommendation, engagement in activities and levels of completion are being monitored, replacement activities can be automatically suggested from a list of pre-defined activities that could be developed with people living with dementia and carers at earlier stages of the disease. Given our participants’ fear and anxiety in relation to certain activities, it will be important for systems to explain why such alternatives are being suggested. The difficulty arises when interests are so unique to the individual and finding replacements that are adequate and effective becomes challenging, as was observed with PLwD5 who could not find a suitable replacement for running. More observational research and longitudinal studies are needed to determine how engagement with technology changes over time with disease progression^68^ as well as how technology can support meaningful alternative activities.

### Limitations

Recruitment for this study took place during COVID-19 when some restrictions were still in place, which made recruitment challenging, both in terms of engaging with HPs to recruit participants and identifying persons living with dementia/carer dyads willing to take part. All of our participants were white Irish. With regard to diversity and representation among the sample selected, the most recent census carried out in Ireland estimated the percentage of white Irish to be 77% of the total population. The next largest group in terms of ethnicity was “any other white background” (10%), followed by Indian/Pakistani/Bangladeshi (2%), and Black or Black Irish (1%).^77^ Given that migration to Ireland is a relatively recent trend, it stands to reason that the percentage of white Irish is considerably higher among the older population. For this reason, the sample can be considered broadly representative of the older population in Ireland. While every effort was made to recruit female dementia participants, our sample consisted of all males while all carers were female. Prevalence of dementia is higher in women than in men, women have a higher risk of developing dementia and are also more likely to have more severe symptoms than men.^78,79^ Gender factors can also result in differences between how men and women experience and cope with dementia. For example, women can find the shift in identity from carer to being cared for as very difficult, while willingness to accept support can also be different for men and women.^78^ It is also understood that gender influences shared decision making, although there are discrepancies across studies as to whether men or women are more likely to be involved in shared decision making.^74^ Furthermore, women are more likely to act as carers for people with dementia.^78^ This means that interventions for people with dementia and their carers, including digital interventions, may need to be tailored based on gender. Indeed, HCI research has shown gender-based differences in technology use and behavior.^80^ The next phase of our research (outlined in the following section) will seek to rectify this issue by actively recruiting female dementia male carer dyads. A further limitation is that the sample size of each individual participant group might be considered small. However, a multistakeholder approach was taken providing a range of perspectives and significant amounts of data were collected, sometimes across multiple interview or focus group sessions. It should also be noted that informed consent with this cohort can result in exclusion in certain instances that could be against an individual's wishes.

## Conclusion and future work

People living with dementia, particularly those in the mild to moderate stages, can and should be at the center of their own care-planning and decision-making, afforded the dignity of collaboration and meaningful input where possible. The findings presented in this paper represent a multi-stakeholder view of the relationship between engaging in daily and meaningful activities, quality of life, and living well with dementia, with perspectives sought and feedback analyzed from people living with mild dementia, informal carers, and HPs working in this area. The thematic analysis allowed for the identification of three main themes: maintaining a sense of purpose and identity, learned helplessness, and shared decision-making and collaboration. The findings informed a set of design recommendations that may be of value to those within the HCI community, with accessible education, collaborative care-planning and goal-setting, and the need for designs to consider disease progression key areas to reflect on when designing for dyads. The next phase of our research will extend the findings presented here through engaging dyads in co-design workshops with the aim of co-designing and developing a technology toolkit that reflects the design recommendations provided in this article.

## Supplemental Material

sj-jpg-1-dhj-10.1177_20552076231222427 - Supplemental material for Designing technology to support greater participation of people living with dementia in daily and meaningful activities Click here for additional data file. Supplemental material, sj-jpg-1-dhj-10.1177_20552076231222427 for Designing technology to support greater participation of people living with dementia in daily and meaningful activities by Michael Wilson, Julie Doyle, Jonathan Turner, Ciaran Nugent and Dympna O’Sullivan in DIGITAL HEALTH

sj-docx-2-dhj-10.1177_20552076231222427 - Supplemental material for Designing technology to support greater participation of people living with dementia in daily and meaningful activities Click here for additional data file. Supplemental material, sj-docx-2-dhj-10.1177_20552076231222427 for Designing technology to support greater participation of people living with dementia in daily and meaningful activities by Michael Wilson, Julie Doyle, Jonathan Turner, Ciaran Nugent and Dympna O’Sullivan in DIGITAL HEALTH

sj-docx-3-dhj-10.1177_20552076231222427 - Supplemental material for Designing technology to support greater participation of people living with dementia in daily and meaningful activities Click here for additional data file. Supplemental material, sj-docx-3-dhj-10.1177_20552076231222427 for Designing technology to support greater participation of people living with dementia in daily and meaningful activities by Michael Wilson, Julie Doyle, Jonathan Turner, Ciaran Nugent and Dympna O’Sullivan in DIGITAL HEALTH
