# Supplementary figures and images for: Designing technology to support greater participation of people living with dementia in daily and meaningful activities
Source: Digit Health. 2024 Jan 15;10:20552076231222427. doi: 10.1177/20552076231222427 (PMC10793193; doi:10.1177/20552076231222427)

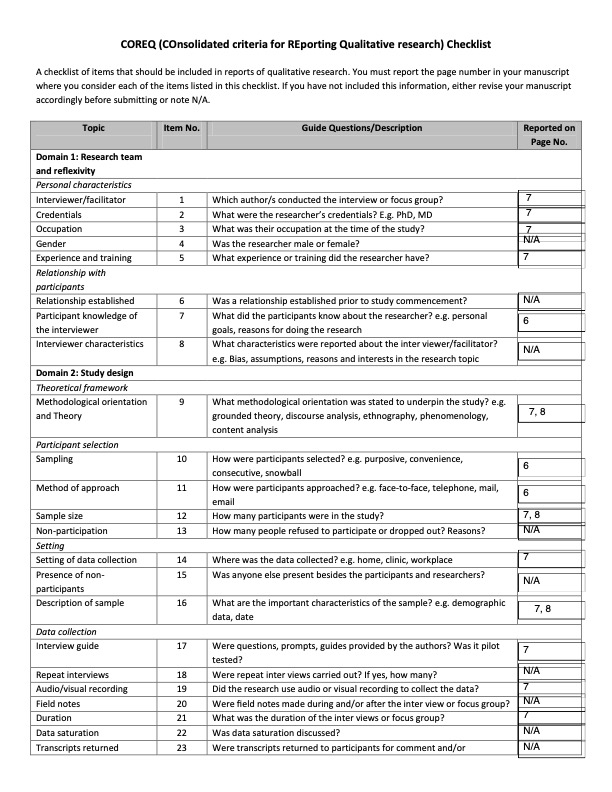

Supplement: sj-jpg-1-dhj-10.1177_20552076231222427 - Supplemental material for Designing technology to support greater participation of people living with dementia in daily and meaningful activities [file sj-jpg-1-dhj-10.1177_20552076231222427.jpg]
