# Supplementary material for: Designing technology to support greater participation of people living with dementia in daily and meaningful activities
Source: Digit Health. 2024 Jan 15;10:20552076231222427. doi: 10.1177/20552076231222427 (PMC10793193; doi:10.1177/20552076231222427)
Supplement: sj-docx-2-dhj-10.1177_20552076231222427 - Supplemental material for Designing technology to support greater participation of people living with dementia in daily and meaningful activities [file sj-docx-2-dhj-10.1177_20552076231222427.docx]

**Smart Dementia Care – Preliminary Interview Protocol (Carers)**

Duration: 40 minutes to 1 hour - can split into two 30 minute sessions if needed Participants: Informal carers of people living with dementia
Format/Location: Online video call (preferable), or over the phone

Notes/Comments: Communicate to participants clearly that they should not feel under any pressure to discuss any issues or topics they are not comparable with. Discussion should be as informal and friendly as possible, with follow-up answers and further topic/areas of discussion welcome. Encourage discussion and answers to ‘Why’. Explain that the conversation can be paused for beaks or ended at any point, should they so wish. Make clear that the conversation will be recorded, and privacy and anonymity will be ensured.

Main tasks (overview):

1. General health and wellbeing: parameters of health that are most important to people

living with dementia

1. Approaches to health self-management and techniques currently employed for

managing overall wellbeing

1. Quality of life and goals/hopes for the future
2. Instrumental activities of daily living
   1. Shopping
   2. Transportation
   3. Housework
   4. Managing finances
   5. Meal preparation
   6. Managing communication
   7. Managing medication
3. Meaningful activities

**Section 1 (if splitting session into two) Topic 1:** General health and wellbeing

What do you think are the most important areas of health and wellbeing for people living with dementia? Why? *Prompt social interaction, cognitive training, mobility, sleep, mood, disease management, blood pressure etc.*


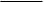

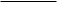

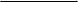

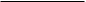

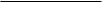


How would you describe your care recipient’s (CR) health and wellbeing overall? Does he/she have any other conditions or health concerns apart from dementia? **Note: check relationship of CR (e.ge. husband, mother) and then use that from here on*

Describe your average day. Do you each have a routine? At what parts of the day do you feel your CR is at his/her most active? Is it important to have regular patterns? Why?

How important is physical activity and exercise for your CR? Would you want to know if he/she is staying active/getting exercise? Why/why not? If you had this type of information, what do you think you could both do with it?

*(If time:* Are there days when he/she walks more than others? How much time would he/she spend inside vs outside the home? )

*(If time*: How well do you feel he/she sleeps in general? Do you think sleep quality is important for overall wellbeing?)

**Topic 2:** Approaches to maintaining good health and wellbeing

Are there any techniques that you use to help your CR manage his/her own health and wellbeing? Does he/she use memory aids, visual clues or reminders? If so, how? If not, would you consider this?

Has a doctor or nurse ever advised you on what you can do to help your CR to look after his/her health at home?

Are there any challenges that you face in looking after your CR’s health and wellbeing day to day? Do you have any support? Are there any supports you can think of that you feel could assist you both?

Some people like to set goals in relation to their health. For example, this could be a goal to remember to take medication as prescribed, or a goal to get a certain amount of physical activity each day or week. Do you and your CR set goals? Do you think that setting some goals might be useful to you both?

Do you use any technologies at home for looking after your CR’s health and wellbeing (*provide examples – smartphones/tablets, reminders, wearable trackers, sensors).* Do you think technologies like these could be useful to you both? How?

**Topic 3 -** Quality of life and goals/hopes for the future

What does having a ‘good’ quality of life mean to you? How can this be maintained or improved? What are your hopes and goals for your CR in terms of living well in the future?
Which areas of daily life do you think your CR would want to maintain as much as possible?

**Section 2 (if splitting session into two) Topic 4:** Instrumental activities of daily living

I’d like to ask you some questions about everyday activities and how you and your CR do or do not approach them.

**Where relevant - check on carer’s own role with these activities*

Instrumental activities of daily living

1. transportation (manage transportation either through driving or other means),
a. How does he/she get from place to place? Does he/she encounter any difficulties? Do you drive? Does your CR drive? Does he/she ever use public transport? Where/When? If not, why not? How does he/she find using it? What

would make it easier?
2. shopping (ability to procure groceries and other necessary items),

a. In a normal week, who looks after shopping in the household? How often do you (or CR/both) go shopping? Where/When? Do either of you shop online? For what? Does your CR face any difficulties when shopping? Do you? What would make it easier?

3. managing finances (ability to pay bills, manage bank balance and take care of financial assets),

a. Who manages finances in the household? How are bills paid? Does your CR handle cash? Does he/she use contactless/cards? How does he/she check his/her bank balance and carry out everyday/normal banking tasks? Does he/she use online banking? If so, what are your experiences with it? Does he/she face any difficulties managing finances in general? Do you? What would make it easier?

4. meal preparation (connected to shopping and involves everything necessary to put regular meals together),

a. Who looks after meal preparation in the household? Do you think it important to look after your diet? How? Does your CR eat at regular mealtimes? What challenges could he/she face when preparing meals? Does he/she use recipes or

other supports? (Do you?) Would he/she use the internet as a source of

information for cooking/diet advice? (Would you?)
5. housekeeping and home maintenance (cleaning up after meals, maintaining a clean and

tidy home in general, doing laundry, keeping up with home maintenance),
a. Who looks after housekeeping tasks in the home such as cleaning up after meals, doing laundry and tidying/cleaning? Which tasks are particularly challenging?

What would make it easier?
6. managing communication (ability to use telephone and mail to communicate and keep

in touch with others),
a. How does your CR keep in touch with family/friends? How does he/she access

services such as GP, pharmacy, utilities or government services such as Revenue etc.? What challenges does he/she face with services moving from post/telephone to online? Does your CR own and use a smartphone/tablet/PC or laptop? If so, how often does he/she use it? (What about you?) Are there any challenges he/she faces using smartphones and the internet in general? (What about you?) What would his/her preference be for maintaining contact with others going forward?

7. managing medications (ability to procure medications when required, taking correct dosage as advised)

a. If your CR is taking medication, how does he/she go about buying/obtaining and storing? Does he/she face any challenges managing medication? Does he/she use any memory aids or reminders to keep track? What could make this aspect of your life easier?

**Topic 5:** Meaningful Activities

Thinking about an average day, what activities do you think feel meaningful for your CR? What brings him/her satisfaction? What would he/she miss doing, even small activities? *Provide examples such as gardening (planting flowers, weeding, watering plants), repair/upkeep work (painting furniture, hanging pictures) physical exercise (going for a walk) or hobbies (playing an instrument, listening to music, drawing, painting, collecting etc..).*

What does he/she look forward to doing on a normal day?

Next, I’d like to talk about some areas of life that might be important to you and your CR. For each, I’ll ask you how important they are for your CR, both right now but also thinking about the future.

- ●  Physical activity and exercise
- ●  Household chores
- ●  Leisure activities – *examples*?
- ●  Hobbies and personal interest – *examples*?
- ●  Social activities, community involvement

Think about the following activities. How meaningful, important, or personally satisfying are they for your CR? Which would he/she particularly like to continue in the future?

- ●  Music and entertainment (watching movies, listening to music, playing instruments)
- ●  Cognitive activities such as puzzles or card games
- ●  Arts and crafts (painting or woodwork etc.)
- ●  Manipulation activities (organizing, sorting)
- ●  Family/social reminiscence (photo albums)
- ●  Socializing (*social networks?)*
- ●  General domestic activities such as laundry or preparing snacks

Does he/she have any preferences in terms of maintaining/keeping up these activities in the future?

Are there any other activities you can think of that have not been mentioned?

**Smart Dementia Care – Preliminary Interview Protocol**

Duration: 40 minutes to 1 hour - can split into two 30 minute sessions if needed Participants: People living with early-stage dementia
Format/Location: Online video call (preferable), or over the phone

Notes/Comments: Communicate to participants clearly that they should not feel under any pressure to discuss any issues or topics they are not comparable with. Discussion should be as informal and friendly as possible, with follow-up answers and further topic/areas of discussion welcome. Encourage discussion and answers to ‘Why’. Explain that the conversation can be paused for beaks or ended at any point, should they so wish. Make clear that the conversation will be recorded, and privacy and anonymity will be ensured.

Main tasks (overview):

1. General health and wellbeing: parameters of health that are most important to

people living with dementia

1. Approaches to health self-management and techniques currently employed for

managing overall wellbeing

1. Quality of life and goals/hopes for the future
2. Instrumental activities of daily living
   1. Shopping
   2. Transportation
   3. Housework
   4. Managing finances
   5. Meal preparation
   6. Managing communication
   7. Managing medication
3. Meaningful activities

**Section 1 (if splitting session into two) Topic 1:** General health and wellbeing

What do you think are the most important areas of health and wellbeing for people living with dementia? Why? *Prompt social interaction, cognitive training, mobility, sleep, mood, disease management, blood pressure etc.*

How would you describe your health and wellbeing overall? Do you have any other conditions or health concerns apart from dementia?


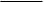

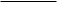

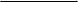

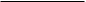

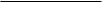


Describe your average day. Do you have a routine? At what parts of the day do you feel you are at your most active? Is it important to have regular patterns?

How important is physical activity and exercise for you? Would you want to know how many steps you take on a given day? Why/why not? If you had this type of information, what do you think you could do with it?

*(If time:* Are there days when you walk more than others? How much time would you spend inside vs outside the home? )

*(If time*: How well do you feel you sleep in general? Do you think sleep quality is important for overall wellbeing?)

**Topic 2:** Approaches to maintaining good health and wellbeing
Are there any techniques that you use to manage your own health and wellbeing? Do you

use memory aids, visual clues or reminders? If so, how? If not, would you consider this?

Has a doctor or nurse ever advised you on what you can do at home to look after your health?

Are there any challenges that you face in looking after your health and wellbeing day to day? Do you have any support? Are there any supports you can think of that you feel could assist you?

Some people like to set goals in relation to their health. For example, this could be a goal to remember to take medication as prescribed, or a goal to get a certain amount of physical activity each day or week.Do you set goals? Do you think that setting some goals might be useful to you?

Do you use any technologies at home for looking after your health and wellbeing (*provide examples – smartphones/tablets, reminders, wearable trackers, sensors).* Do you think technologies like these could be useful to you? How?

**Topic 3 -** Quality of life and goals/hopes for the future

What does having a ‘good’ quality of life mean to you? How can this be maintained or improved? What are your hopes and goals for living well in the future?
Which areas of daily life do you want to maintain as much as possible?

**Section 2 (if splitting session into two) Topic 4:** Instrumental activities of daily living

I’d like to ask you some questions about everyday activities and how you do or do not approach them.

Instrumental activities of daily living

1. transportation (manage transportation either through driving or other means),
a. How do you get from place to place? Do you encounter any difficulties? Do you drive? Do you ever use public transport? Where/When? If not, why not?

How do you find using it? What would make it easier?
2. shopping (ability to procure groceries and other necessary items),

a. In a normal week, who looks after shopping in your household? How often do you go shopping? Where/When? Do you shop online? For what? Do you face any difficulties when shopping? What would make it easier?

3. managing finances (ability to pay bills, manage bank balance and take care of financial assets),

a. Who manages finances in your household? How are bills paid? Do you handle cash or use contactless/cards mainly? How do you check your bank balance and carry out everyday/normal banking tasks? Do you use online banking? If so, what are your experiences with it? Do you face any difficulties managing finances in general? What would make it easier?

4. meal preparation (connected to shopping and involves everything necessary to put regular meals together),

a. Who looks after meal preparation in our household? Do you think it important to look after your diet? How? Do you eat at regular mealtimes? What challenges could you face when preparing meals? Do you use recipes or other supports? Would you use the internet as a source of information for cooking/diet advice?

1. housekeeping and home maintenance (cleaning up after meals, maintaining a clean and tidy home in general, doing laundry, keeping up with home maintenance),

a. Who looks after housekeeping tasks in the home such as cleaning up after meals, doing laundry and tidying/cleaning? Which tasks are particularly challenging? What would make it easier?

1. managing communication (ability to use telephone and mail to communicate and keep in touch with others),

a. How do you keep in touch with family/friends? How do you access services such as GP, pharmacy, utilities or government services such as Revenue etc.? What challenges do you face with services moving from post/telephone to online? Do you own and use a smartphone/tablet/PC or laptop? If so, how often do you use it? Are there any challenges you face using smartphones and the internet in general? What would your preference be for maintaining contact with others going forward?

7. managing medications (ability to procure medications when required, taking correct dosage as advised)

a. If you are taking medication, how do you go about buying/obtaining and storing? Do you face any challenges managing your medication? Do you use any memory aids or reminders to keep track? What could make this aspect of your life easier?

**Topic 5:** Meaningful Activities

Thinking about an average day, what activities feel meaningful for you? What brings you satisfaction? What would you miss doing, even small activities? *Provide examples such as gardening (planting flowers, weeding, watering plants), repair/upkeep work (painting furniture, hanging pictures) physical exercise (going for a walk) or hobbies (playing an instrument, listening to music, drawing, painting, collecting etc..).*

What do you look forward to doing on a normal day?

Next, I’d like to talk about some areas of life that might be important to you. For each, I’ll ask you how important they are for you, both right now but also thinking about the future.

- ●  Physical activity and exercise
- ●  Household chores
- ●  Leisure activities – *examples*?
- ●  Hobbies and personal interest – *examples*?
- ●  Social activities, community involvement

Think about the following activities. How meaningful, important, or personally satisfying are they for you? Which would you particularly like to continue in the future?

- ●  Music and entertainment (watching movies, listening to music, playing instruments)
- ●  Cognitive activities such as puzzles or card games
- ●  Arts and crafts (painting or woodwork etc.)
- ●  Manipulation activities (organizing, sorting)
- ●  Family/social reminiscence (photo albums)
- ●  Socializing (*social networks?)*
- ●  General domestic activities such as laundry or preparing snacks

Do you have any preferences in terms of maintaining/keeping up these activities in the future?

Are there any other activities you can think of that have not been mentioned?

**Smart Dementia Care – Preliminary Interview Protocol (Healthcare Professionals)**

Duration: 40 minutes to 1 hour - can split into two 30 minute sessions if needed Participants: Healthcare professionals working with people living with dementia Format/Location: Online video call (preferable), or over the phone

Notes/Comments: Communicate to participants clearly that they should not feel under any pressure to discuss any issues or topics they are not comparable with. Discussion should be as informal and friendly as possible, with follow-up answers and further topic/areas of discussion welcome. Encourage discussion and answers to ‘Why’. Explain that the conversation can be paused for beaks or ended at any point, should they so wish. Make clear that the conversation will be recorded, and privacy and anonymity will be ensured.

Main tasks (overview):

1. General health and wellbeing: parameters of health that are most important to people

living with dementia

1. Approaches to health self-management and techniques currently employed for

managing overall wellbeing

1. Quality of life and goals/hopes for the future
2. Instrumental activities of daily living
   1. Shopping
   2. Transportation
   3. Housework
   4. Managing finances
   5. Meal preparation
   6. Managing communication
   7. Managing medication
3. Meaningful activities

**Section 1 (if splitting session into two) Topic 1:** General health and wellbeing


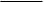

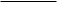

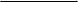

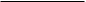

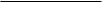


What do you think are the most important areas of health and wellbeing for people living with dementia? Why? *Prompt social interaction, cognitive training, mobility, sleep, mood, disease management, blood pressure etc.*

What are some common conditions or health concerns service users frequently have apart from dementia?

Describe a typical day interacting with service users. Do you have a routine? At what parts of the day do you feel someone living with early stage dementia is at his/her most active? Is it important for them to have regular patterns? Why?

How important is physical activity and exercise for PwD? Would you want to know if he/she is staying active/getting exercise? Why/why not? If you had this type of information, what do you think you could both do with it?

**Topic 2:** Approaches to maintaining good health and wellbeing

Are there any techniques that you use to help your service users manage their own health and wellbeing? Do you use memory aids, visual clues or reminders? If so, how? If not, would you consider this?

What advice would you typically give to someone living with dementia to help him/her look after his/her health at home? **Use of a care plan etc.*

Can you think of any common challenges that PwD face in looking after their health and wellbeing day to day? Are there any supports you can think of that you feel could assist someone living with dementia? **local services, support groups, internet resources, technology solutions*

Some people like to set goals in relation to their health. For example, this could be a goal to remember to take medication as prescribed, or a goal to get a certain amount of physical activity each day or week. Do your service users typically set goals? Do you think that setting some goals might be useful to someone living with dementia?

Do you know of any technologies someone living with dementia can use at home for looking after their health and wellbeing (*provide examples – smartphones/tablets, reminders, wearable trackers, sensors).* Do you think technologies like these could be useful? How?

**Topic 3 -** Quality of life and goals/hopes for the future
What does having a ‘good’ quality of life mean for a person with dementia? How can this be maintained

or improved? Is this something you discuss with the person as part of their care plan? What are common hopes and goals for PwD in terms of living well in the future?
Which areas of daily life do you think PwD would want to maintain as much as possible?

**Section 2 (if splitting session into two) Topic 4:** Instrumental activities of daily living

I’d like to ask you some questions about everyday activities and how someone living with dementia may or may not approach them. Do you address any of these activities as part of your care? How?

**Where relevant - check on carer’s own role with these activities*

Instrumental activities of daily living

1. transportation (manage transportation either through driving or other means),
a. How might he/she get from place to place? Would he/she encounter any difficulties? Might he/she drive? Is a CR more likely to drive? Would he/she ever use public transport? Where/When? If not, why not? How might he/she find

using it? What would make it easier?
2. shopping (ability to procure groceries and other necessary items),

a. In a normal week, who generally looks after shopping in a household? How often would he/she (or CR/both) go shopping? Where/When? Would someone living with dementia shop online? For what? Would someone living with dementia face any difficulties overall when shopping? What would make it easier?

3. managing finances (ability to pay bills, manage bank balance and take care of financial assets),

a. Who typically manages finances in a household? How are bills paid? Would he/she handle cash? Would he/she use contactless/cards? How might he/she check his/her bank balance and carry out everyday/normal banking tasks? Would he/she use online banking? Would he/she face any difficulties managing finances in general? What would make it easier?

4. meal preparation (connected to shopping and involves everything necessary to put regular meals together),

a. Who generally looks after meal preparation in a household? How important is it for PwD to look after their diet? How? Would PwD typically eat at regular mealtimes? What challenges could he/she face when preparing meals? Would he/she use recipes or other supports? Would he/she use the internet as a source of information for cooking/diet advice?

1. housekeeping and home maintenance (cleaning up after meals, maintaining a clean and tidy home in general, doing laundry, keeping up with home maintenance),

a. Who typically looks after housekeeping tasks in the home such as cleaning up after meals, doing laundry and tidying/cleaning? Which tasks are particularly challenging? What would make it easier?

1. managing communication (ability to use telephone and mail to communicate and keep in touch with others),

a. How do PwD typically keep in touch with family/friends? How would he/she access services such as GP, pharmacy, utilities or government services such as Revenue etc.? What challenges might he/she face with services moving from post/telephone to online? In your experience, do PwD own and use a smartphone/tablet/PC or laptop? If so, how often would he/she use it? Are there any challenges he/she might face using smartphones and the internet in general? What would his/her preference be for maintaining contact with others going forward?

7. managing medications (ability to procure medications when required, taking correct dosage as advised)

a. If someone living with dementia is taking medication, how might he/she go about buying/obtaining and storing? Would he/she face any challenges managing medication? Do you find many PwD using memory aids or reminders to keep track? What could make this aspect of life easier?

**Topic 5:** Meaningful Activities

Thinking about an average day, what activities do you think feel meaningful for someone living with dementia? What brings him/her satisfaction? What would he/she miss doing, even small activities? *Provide examples such as gardening (planting flowers, weeding, watering plants), repair/upkeep work (painting furniture, hanging pictures) physical exercise (going for a walk) or hobbies (playing an instrument, listening to music, drawing, painting, collecting etc..).*

What might he/she look forward to doing on a normal day?

How important do you as a healthcare professional feel meaningful activities are for people with dementia and their carers? Is this something you ask service users about? Would this be part of their care plan?

Next, I’d like to talk about some areas of life that might be important to PwD in general. For each, I’ll ask you how important they are, both right now but also thinking about the future.

- ●  Physical activity and exercise
- ●  Household chores
- ●  Leisure activities – *examples*?
- ●  Hobbies and personal interest – *examples*?
- ●  Social activities, community involvement

Think about the following activities. How meaningful, important, or personally satisfying are they for PwD in general? Which might he/she particularly like to continue in the future?

- ●  Music and entertainment (watching movies, listening to music, playing instruments)
- ●  Cognitive activities such as puzzles or card games
- ●  Arts and crafts (painting or woodwork etc.)
- ●  Manipulation activities (organizing, sorting)
- ●  Family/social reminiscence (photo albums)
- ●  Socializing (*social networks?)*
- ●  General domestic activities such as laundry or preparing snacks

Do you think someone living with dementia might have any preferences in terms of maintaining/keeping up these activities in the future?

Are there any other activities you can think of that have not been mentioned?
