# Supplementary material for: Designing technology to support greater participation of people living with dementia in daily and meaningful activities
Source: Digit Health. 2024 Jan 15;10:20552076231222427. doi: 10.1177/20552076231222427 (PMC10793193; doi:10.1177/20552076231222427)
Supplement: sj-docx-3-dhj-10.1177_20552076231222427 - Supplemental material for Designing technology to support greater participation of people living with dementia in daily and meaningful activities [file sj-docx-3-dhj-10.1177_20552076231222427.docx]

This file outlines a number of examples to demonstrate the coding process applied to the transcripts from the interviews and focus groups. Within each example, a number of quotes from transcripts are provided outlining how quotes (in red) were coded initially (in blue) and then clustered, followed by categorization into sub-themes and themes.

**Quotes:**

*Do you cook James? P5: No, no, that has been taken out of my hands* (Effect of disease on ability to do things you want to do)

*you know*

*P2: there are a lot of things I want to get going for a while now and I just wasn’t capable of doing it… Well I lost mobility and the interest* (Effect of disease on ability to do things you want to do) *in doing the google searching. I just got lost a bit* (Effect of disease on ability to do things you want to do) *as I said I had a bad experience and I kind of lost and then this problem it has hit me* (Effect of disease on ability to do things you want to do)

*P1: We were going to the pub for a few drinks.* (Effect of disease on ability to do things you want to do) *Helena wasn’t going and I told my brothers, I reluctantly went. I was terrified.* (Effect of disease on ability to do things you want to do) *My brother asked why are you so afraid I said I don’t know,*

**Quotes:**

*HP 1: If your job was always kettle, make the porridge and the other person set the table. That task should remain the same.*  (Household Roles and Feeling Needed)

*HP 1: Who did all the bins, it will always be one person.* (Household Roles and Feeling Needed) *You know, who did the cooking it will always be one person.*

*HP 1: So everyone is half and half like most houses run on half and half and its important find that out.* (Household Roles and Feeling Needed)

*IC 4: it would be joint,* (Household Roles and Feeling Needed) *I mean, laundry I would sort of organise it, I mean Brian would hang out the washing* (Household Roles and Feeling Needed)

*IC 4: Brian would help me prepare* (Household Roles and Feeling Needed) *the vegetables to make the soup.*

*P2: but she does the cooking and if there’s potatoes to be peeled, or something like that, and I will get the carrots ready* (Household Roles and Feeling Needed)

**Quotes:**

*HP 1: It’s a version of the activity that was.* (Link between past interests and QoL)

*HP 3: if there is an area of interest that they used to be interested in* (Link between past interests and QoL) *but stopped being interested in because maybe their symptoms are impacting* (Link between past interests and QoL) *on that and it’s our job then to try and grade the activity to a level they can then return to doing it* (Link between past interests and QoL)

*HP 2: it’s trying to make it personalised* (Link between past interests and QoL) *to see like what is it but equally…, it’s trying to get people to sort of to try something new* (Link between past interests and QoL) *it’s just if you can get them to do that.*

*P2: Yes, I would have been able* (Link between past interests and QoL) *to do all the home maintenance and that sort of thing.*

*P1: So rather than beating myself up about it, I will try and get something to replace it.* (Link between past interests and QoL) *I will try and get something else to look forward to.*

| Category/Code | Sub-theme | Theme |
| --- | --- | --- |
| Assuming new roles  Feeling needed vs feeling useless  Sense of responsibility, independence  Effect of disease on ability to do things you want to do  Condition affecting independence  Loss of responsibility and independence  Maintaining independence  Household Roles and Feeling Needed  Sense of empowerment  Mobility and transport  Daily routine  Boredom and feeling frustrated  Identifying interests  Link between past interests and QoL  Finding meaning  Appreciating the here and now | **Impact of dementia on sense of self**  **Strategies for maintaining purpose**  **Identifying future meaningful activities** | **Maintaining sense of purpose and identity** |
